# Editorial: Evaluation of the left atrium: Its role in atrial fibrillation and diastolic function

**DOI:** 10.3389/fcvm.2023.1130531

**Published:** 2023-02-21

**Authors:** Liza Thomas, Kazuaki Negishi, Faraz Khalid Pathan

**Affiliations:** ^1^Department of Cardiology, Westmead Hospital, Westmead, NSW, Australia; ^2^Westmead Clinical School, University of Sydney, Sydney, NSW, Australia; ^3^South West Clinical School, University of New South Wales, Kensington, NSW, Australia; ^4^Department of Cardiology, Nepean Hospital, Nepean, NSW, Australia; ^5^Nepean Clinical School, University of Sydney, Sydney, NSW, Australia

**Keywords:** left atrium, atrial strain, left atrial size, prognostic value, biomarker

This special issue covers an emerging clinical area, “Evaluation of the left atrium: its role in atrial fibrillation and diastolic function”. The left atrium is considered as the HbA1c for diastolic function i.e., a surrogate marker of severity and chronicity of diastolic dysfunction. Its importance is reflected in the incorporation of left atrial (LA) volume in the guidelines for evaluation of diastolic function (1). However, evaluation of LA function, specifically with the emergence of strain analysis (2), has demonstrated that LA strain is altered even prior to LA enlargement (3), and incorporation of LA strain reclassified those with indeterminate diastolic function (4). The other key areas of utility of LA metrics are atrial fibrillation (AF) (5), stroke (6), and heart failure with preserved ejection fraction (HFpEF) (7). LA strain using echocardiography has demonstrated diagnostic and prognostic utility. However, other imaging modalities including cardiac computerized tomography (CT) and cardiac magnetic resonance (CMR) imaging are emerging useful modalities (8).

In this issue, LA function was evaluated using LA strain in patients with hypertrophic cardiomyopathy (HCM) to determine predictors of future development of AF (Mandes et al.). A total of 126 patients with HCM were followed for a median of 56 months during which time 30.9% developed AF. Importantly, LA booster pump strain and P wave electromechanical dispersion were found to be independent predictors over traditional parameters, such as, LA diameter and LA volume. An exciting finding in a subgroup with normal sized LA was that P wave electromechanical dispersion was still a predictor of AF.

Studies in AF patients undergoing catheter ablation demonstrated that LA strain predicts AF recurrence after ablation. However, this was mostly in patients with paroxysmal AF with LA strain measured in sinus rhythm, and little is known about LA strain during AF. An original study in this issue of the *journal* evaluated whether LA strain during persistent AF could predict (1) atrial fibrosis measured by low voltage area (LVA) and (2) AF recurrence after catheter ablation (Marchandise et al.). The study found that LA strain and LA stiffness index (E/e'/LA strain) were independently associated with LVA. Both LA strain and LA stiffness index had incremental predictive value for AF recurrence over risk stratification schemes (CHARGE-AF or CHA2DS2-VASc score). LA strain could further risk stratify recurrence even during AF rhythm.

A consequence of AF is the development of LA appendage thrombus, a recognized cause of cardio embolic stroke. While transesophageal echocardiography (TOE) has hitherto been the modality of choice for detection of LA thrombus, cardiac CT is an emerging modality, as was demonstrated during the recent COVID pandemic when TOE was considered a high-risk procedure for transmission of COVID. Due to slow flow in the LA appendage, false positives and a low positive predictive value has historically been an impediment, one that was addressed by multiple delayed phase imaging trials (9). The current study builds upon previous studies which improved specificity of CT to exclude LA appendage thrombus (Li et al.). The authors investigated the role of multi-time point delayed CT including 1 min and 3 min delayed imaging. The rate of false positives was very high in the arterial phase (46%) and even at 1 min delayed-phase was modest (7%). The 3^rd^ time point at 3 min was crucial, improving the positive predictive value from 0.57 to 1.00.

Continuing along the same vein of LA appendage thrombi, was a small study that examined the utility of morphological and haemodynamic characteristics of the LA appendage using CT, in AF patients with and without a history of stroke (Wang et al.). 3-dimensional models were reconstructed using CT images and haemodynamic characteristics evaluated using computational fluid dynamics (CFD). The blood residual ratio, and the particle residual ratio were determined. LA appendage (length and actual depth), appendage mouth cross sectional axes, LA and appendage volume and surface area were obtained. Patients with prior stroke demonstrated smaller actual and direct LA appendage length. The blood flow renewal process demonstrated that the residual ratios in both AF groups were high, suggesting that thrombus formation is increased in both groups, though in the AF with stroke group, a lower blood residual ratio was observed, that correlated with the shorter LA appendage length in this group.

The above reports highlight the clinic utility of LA evaluation in cardiomyopathies, AF and stroke. However, attention must be given to obtaining LA measurements to improve the diagnostic and prognostic utility. Evaluation of LA size may be limited on transthoracic echocardiography as it is a far field structure, and in ~8–10% of individuals adequate images and measurements cannot be obtained. Imaging windows are not an issue with cardiac CT or CMR, though their downside is cost and exposure to radiation. For evaluation of LA strain, optimized 2D images need to be obtained at high frame rates (~55 fps). Dedicated LA strain packages are being developed, but the large volume of existing data on LA strain has been obtained using a LV strain package. Measuring LA strain in AF may be challenging and less accurate. While data from few small studies suggest potential usefulness of LA strain, often very low values of LA strain (<10%) are obtained. Thus, at present, AF represents a modest limitation to use LA strain in this setting. An average of 3 beats is recommended in sinus rhythm, and in AF either an average of 3 beats with similar R-R interval or an average of at least 5 beats. Further, automation of measurements as well as inclusion of LA strain measurements in guidelines, may increase uptake as the time involved with manual tracing is a barrier to its use in routine clinical practice.

The lack of standardization has been the Achilles' heel of echocardiography and while the relatively low cost and wide availability are its strengths, has had limited utility in clinical trials as a biomarker. The Palma Echo Platform (PEP) explores the utility of a Core echocardiography laboratory for characterization of cardiac structural and functional abnormalities. The PEP will evaluate a subgroup of 565 individuals (total of 6,874 individuals), with echocardiographic studies performed at the 3 study sites with central evaluation at the PEP core laboratory, to obtain data in a reliable and reproducible manner (López et al.).

In summary, LA metrics of size and function obtained by echocardiography have demonstrated significant clinical utility. However, echocardiography is inherently operator dependent and hence the development of centralized protocols, optimized image acquisition and measurements performed to improve inter-rater variability with reduced test-retest variability will go a long way in the development of echocardiographic metrics as a biomarker for clinical trials. Notwithstanding these issues, LA strain is non-invasive, inexpensive, and without radiation risk with clinical utility to determine the risk of incidental AF and evaluate LV diastolic dysfunction in patients with preserved LVEF and CV risk factors.

## Author contributions

All authors listed have made a substantial, direct, and intellectual contribution to the work and approved it for publication.
